# Coordinated downregulation of Spinophilin and the catalytic subunits of PP1, PPP1CA/B/C, contributes to a worse prognosis in lung cancer

**DOI:** 10.18632/oncotarget.22111

**Published:** 2017-10-26

**Authors:** Eva M. Verdugo-Sivianes, Lola Navas, Sonia Molina-Pinelo, Irene Ferrer, Alvaro Quintanal-Villalonga, Javier Peinado, Jose M. Garcia-Heredia, Blanca Felipe-Abrio, Sandra Muñoz-Galvan, Juan J. Marin, Luis Montuenga, Luis Paz-Ares, Amancio Carnero

**Affiliations:** ^1^ Instituto de Biomedicina de Sevilla (IBIS), Hospital Universitario Virgen del Rocío, Universidad de Sevilla, Consejo Superior de Investigaciones Científicas, Sevilla, Spain; ^2^ CIBER de Cáncer, Instituto de Salud Carlos III, Pabellón 11, Planta 0, Madrid, Spain; ^3^ H120-CNIO Lung Cancer Clinical Research Unit, Instituto de Investigación Hospital 12 de Octubre and CNIO, Madrid, Spain; ^4^ Radiation Oncology Department, Hospital Universitario Virgen del Rocío, Sevilla, Spain; ^5^ Department of Vegetal Biochemistry and Molecular Biology, University of Seville, Seville, Spain; ^6^ Department of Predictive Medicine and Public Health, Universidad de Sevilla, Sevilla, Spain; ^7^ Program in Solid Tumors and Biomarkers, Center for Applied Medical Research (CIMA), Pamplona, Spain

**Keywords:** Spinophilin, PP1, biomarker, lung cancer, therapy

## Abstract

The scaffold protein Spinophilin (Spinophilin, PPP1R9B) is one of the regulatory subunits of phosphatase-1 (PP1), directing it to distinct subcellular locations and targets. The loss of Spinophilin reduces PP1 targeting to pRb, thereby maintaining higher levels of phosphorylated pRb. Spinophilin is absent or reduced in approximately 40% of human lung tumors, correlating with the malignant grade. However, little is known about the relevance of the coordinated activity or presence of Spinophilin and its reported catalytic partners in the prognosis of lung cancer. In the present work, we show that the downregulation of Spinophilin, either by protein or mRNA, is related to a worse prognosis in lung tumors. This effect is more relevant in squamous cell carcinoma, SCC, than in adenocarcinoma. Downregulation of Spinophilin is related to a decrease in the levels of its partners PPP1CA/B/C, the catalytic subunits of PP1. A decrease in these subunits is also related to prognosis in SCC and, in combination with a decrease in Spinophilin, are markers of a poor prognosis in these tumors. The analysis of the genes that correlate to Spinophilin in lung tumors showed clear enrichment in ATP biosynthesis and protein degradation GO pathways. The analysis of the response to several common and pathway-related drugs indicates a direct correlation between the Spinophilin/PPP1Cs ratio and the response to oxaliplatin and bortezomib. This finding indicates that this ratio may be a good predictive biomarker for the activity of the drugs in these tumors with a poor prognosis.

## INTRODUCTION

The *Spinophilin (Spn, PPP1R9b)* gene is located at 17q21.33, a cytogenetic area frequently associated with microsatellite instability and loss of heterozygosity (LOH). LOH in chromosome 17q21.3 has been observed in different tumors, including breast, ovarian, prostate, colorectal, gastric, renal and lung carcinomas as well as in salivary gland carcinosarcoma, an extremely aggressive neoplasm [1–6].

Low levels of Spinophilin expression have been found in lung adenocarcinoma [7], head and neck cancer [8], hepatocellular carcinoma [9], human gastric, small intestine and colorectal adenocarcinoma [10, 11], glioblastoma [12] and breast cancer [13, 14]. In all cases, downregulation of Spinophilin correlated with a higher malignant grade, more aggressive biological behavior and resistance to therapies, leading to faster relapse and poorer patient survival. Furthermore, the loss of Spinophilin also correlated with p53 mutations.

Analysis of human breast tumors showed that Spinophilin downregulation increased the stemness properties and the expression of stem-related genes (Sox2, KLF4, Nanog and OCT4) [14]. Breast tumor stem cells appeared to have low levels of Spinophilin mRNA, and Spinophilin loss correlated with increased stem-like cell appearance in breast tumors, as indicated by an increase in CD44+/CD24- cells. A reduction of the levels of PPP1CA mimicked the cancer stem-like cell phenotype of Spinophilin downregulation, suggesting that the mechanism of Spinophilin involves PP1a [14]. Spinophilin is a scaffolding protein interacting with more than 30 partner proteins, including protein phosphatase 1 (PP1) and F-actin [15–17]. However, the physiological relevance of some of these interactions remains to be determined. Spinophilin performs important functions in the nervous system where it is implicated in regulating spine morphology and density, synaptic plasticity and neuronal migration [15]. Spinophilin also regulates seven-transmembrane receptors and may link these receptors to intracellular mitogenic signaling events that are dependent on p70^S6^ kinase and the small G protein-GEF Rac. Spinophilin also interacts with doublecortin, an actin-binding protein with an established role in the subcellular targeting of PP1. Spinophilin enhances PP1-mediated dephosphorylation of doublecortin [18] concomitant with PP1 localization in the cytosol [19, 20]. Thus, localization of the doublecortin–Spinophilin–PP1 complex in the cytosol inhibits PP1 phosphatase activity, leading to glioma cell death effects via a mitotic spindle catastrophe.

At the functional level, Spinophilin regulates PP1 activity, thereby maintaining higher levels of phosphorylated pRb [21]. This effect contributes to an increase in p53 activity through the increase in ARF protein. However, in the absence of p53, reduced levels of Spinophilin increase the tumorigenic properties of cells. Spinophilin knockout mice have a reduced lifespan, an increased number of tumors and increased cellular proliferation in some tissues, such as the mammary ducts. In addition, the combined loss of Spinophilin and p53 activity in mouse models leads to an increase in mammary carcinomas, confirming the functional relationship between p53 and Spinophilin [22].

The data suggest that the regulation of PP1 activity is the mechanism by which Spinophilin acts to produce its tumor suppressor activity [23]. However, little is known about the relevance of the catalytic subunits of PP1 in cancer prognosis and specifically its coordinated regulation with Spinophilin.

PP1 serine/threonine phosphatases are multimeric enzymes assembled from a small number of catalytic subunits with one of hundreds of regulatory subunits. The regulatory subunit provides precision and specificity to the target [24]. These phosphatase regulators do not share extensive sequence conservation. Instead, they are identified by their physical interaction and function [24]. There are 4 different catalytic PP1 isoforms, PPP1CA/B/C, derived from 3 different genes, plus an alternate splicing of PPP1CC [25–27]. Due to the broad spectrum of activity of each catalytic subunit, not much information regarding their role in tumors or their pathological value has been published. In glioblastoma, PP1A protein expression showed no correlation with prognosis in all cases or on stratification based on IDH1 or ATRX expression. However, nuclear PP1A expression is a strong independent predictor of poor overall survival in p53-positive GBMs only [28]. PPP1CA urinary content was also associated with recurrence in bladder tumors [29]. Finally, analysis of human tumors suggests that one of the PPP1CA alleles might be lost in a high percentage of kidney and colorectal carcinomas [30].

Because the specificity and precision of each PPP1C isoform is given by the regulatory subunit and we detected downregulated Spinophilin in a subgroup of lung tumors, we investigated the effect of Spinophilin regulation of the PP1 catalytic subunits in human lung tumors.

## RESULTS

### Loss of Spinophilin in lung tumors

**Figure 1:**
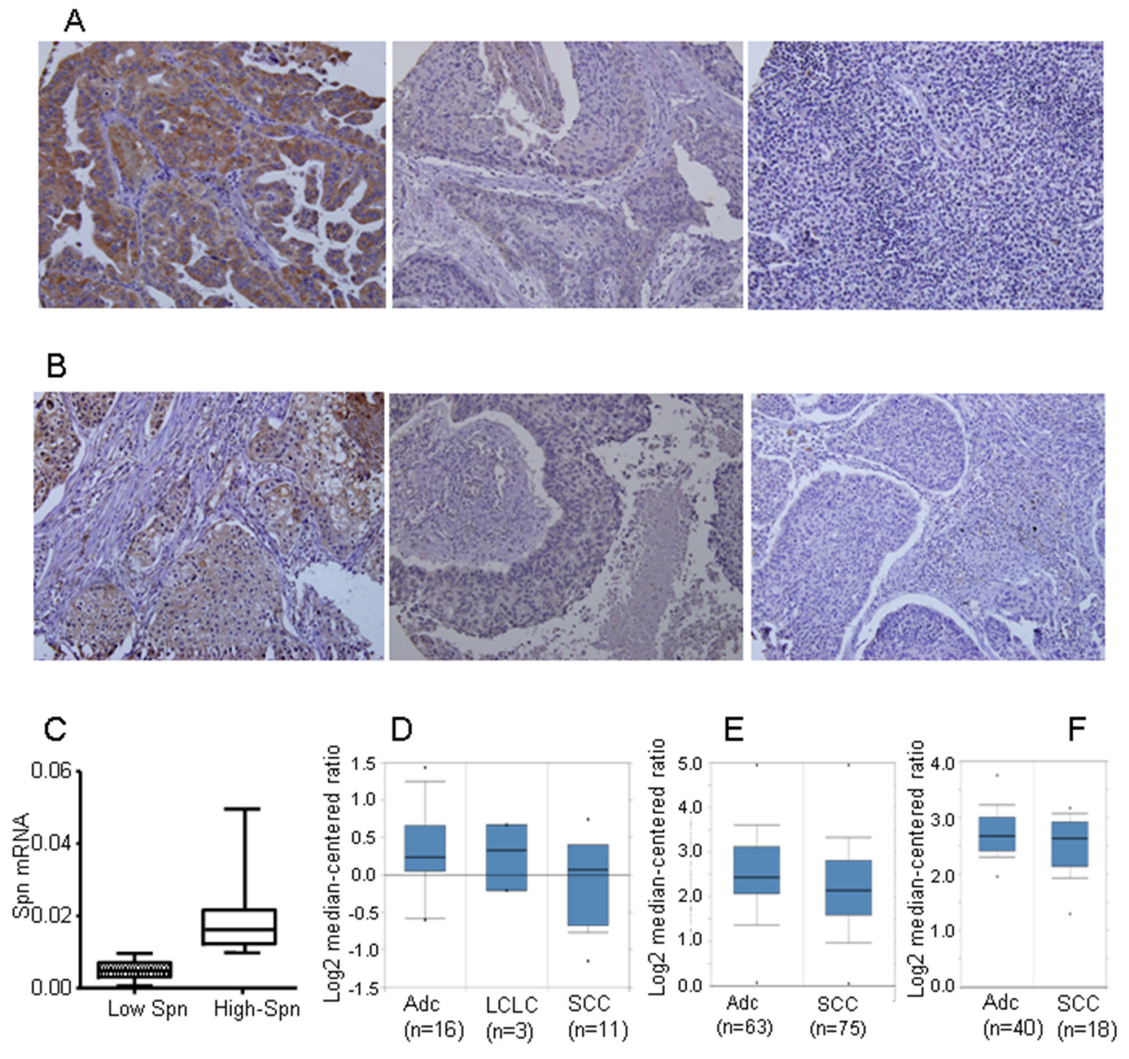
Loss of Spinophilin in human tumors **(A** and **B)** Representative photo of different lung tumors with different Spinophilin levels. **(C)** The levels of Spinophilin mRNA in a cohort of 72 human lung tumors described in reference [7] were analyzed according to the procedure described in M&M. The mean value of mRNA levels of Spinophilin in all samples calculated and tumors distributed according to this mean value. High Spinophilin > mean value; Low Spinophilin > mean value. (D) Analysis of Spinophilin mRNA levels in samples from the cohort of [66]. (E) Analysis of Spinophilin mRNA levels in samples from the cohort of [66]. (E) Analysis of Spinophilin mRNA levels in samples from the cohort of [66]. In all three cases **(D, E** and **F)** the differences of the Spinophilin mRNA levels between ADC and SCC were statistically significant (p<0.05) ADC: Lung Adenocarcinoma; LCLC: Large Cell Lung Carcinoma; SCC: Squamous Cell Lung Carcinoma.

Spinophilin downregulation in human lung tumors is a causal event that triggers an increase in tumorigenic properties, contributing to their malignant status [7]. We have previously described [7, 10] that the cut off for downregulated or lower levels of SPINOPHILIN protein is equal or below 50% of the protein levels found in non-tumoral cells of the same tissue. Downregulation of SPINOPHILIN can be observed by immunostaining (Figure 1A) comprising around 30% samples. On the other hand, we quantified the levels of Spinophilin by mRNA expression levels and detected approximately 30% of tumors with levels of Spinophilin lower than average (= 0.0113) (Figure 1B). This percentage broadly correlated with the percentage of samples showing downregulation of Spinophilin by immunohistochemistry [7]. A more in-depth analysis using public transcriptomic databases shows a similar percentage of tumor samples with downregulated Spinophilin (Supplementary Figure 1). Furthermore, this downregulation of Spinophilin is more pronounced in squamous tumors than in adenocarcinoma (Figure 1C-1E).

A similar percentage of cases showed reduced Spinophilin levels independent of whether the analysis was performed on mRNA or protein, suggesting that Spinophilin downregulation occurs either by the loss of the 17p21 locus or in most cases through the regulation of mRNA levels. To further explore this result, we analyzed Spinophilin promoter methylation in matched lung tumor samples vs. non-tumoral samples from the same patient [31] (Supplementary Table 2). We found that this gene showed increased methylation in tumoral samples vs. non-tumor samples (Table 1). The increased methylation, however, occurs regardless of whether the samples are from adenocarcinoma or squamous carcinoma.

**Table 1:** Methylation of Spinophilin promoter in lung tumors

Spinophilin methylation	NON-tumor	Tumor	Student's *T*-test P value
Adenocarcinoma	0.44 (n=13)	0.52 (n=13)	<0.0001
SCC	0.44 (n=10)	0.51 (n=10)	0.009

The data show the average mean of methylation (see Methods) in the analyzed samples (n=num of samples analyzed).

### Decreased Spinophilin levels predicted poor outcome in lung cancer patients

**Figure 2:**
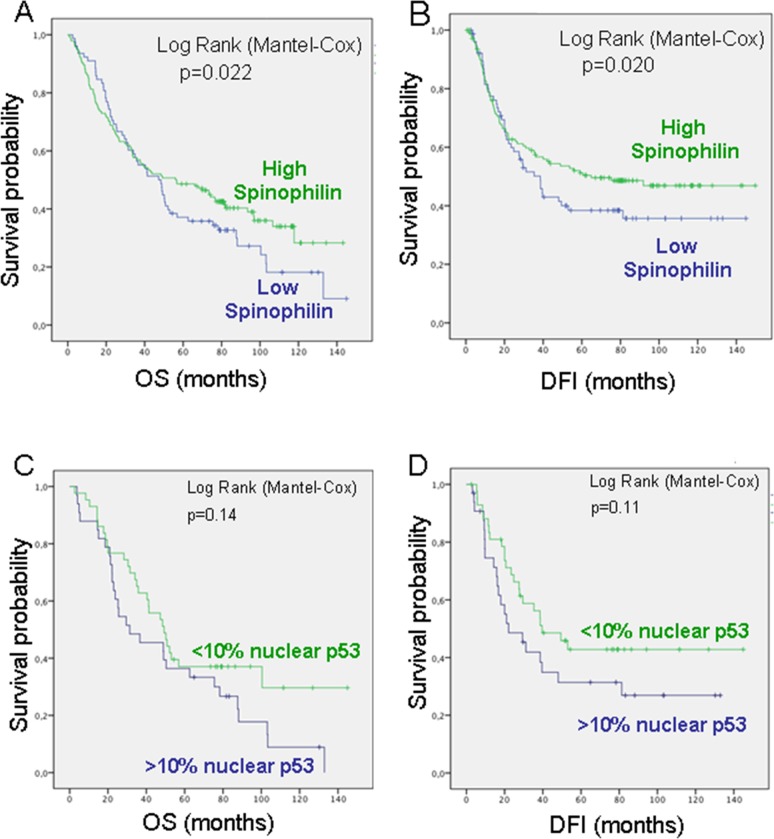
Survival probability of patients with lung cancer according to Spinophilin levels **(A)** Overall survival (OS) and **(B)** Disease-Free Interval (DFI). **(C)** Overall survival (OS) and **(D)** Disease-Free Interval (DFI) in patients with low Spinophilin according to p53 levels.

To evaluate whether SPINOPHILIN levels were associated with clinical outcome, we correlated SPINOPHILIN immunohistochemical staining with patient disease-free interval (DFI) and overall survival (OS). Decreased SPINOPHILIN levels were associated with a poorer OS (p=0,022) and DFI (P=0.020) in patients with lung cancer (Figure 2A and 2B). Multivariate analyses confirmed ECOG and stage as independent predictive factors for PFS. Regarding overall survival, only stage retained independent prognostic significance in the Cox multiple regression model (Supplementary Table 3). The analysis of concurrent molecular alterations showed a correlation with nuclear p53 staining [7]. Therefore, we measured the predictive value of SPINOPHILIN in human lung tumors with nuclear accumulation of p53 (Figure 2C and 2D). We found that low SPINOPHILIN correlated with high levels of nuclear p53 (>10%), as an indicator of mutant p53, and this pattern (low SPINOPHILIN and mutant p53) correlated with a worse prognosis in both OS and DFI (Figure 2C and 2D).

To further assess this hypothesis, we evaluated the association of Spinophilin mRNA levels with tumor response to patient survival in independent cohorts of publicly available databases (Supplementary Figure 1). Low levels of Spinophilin mRNA in lung tumor tissue samples of different databases were always predictive of worse survival probability, correlating with the findings of immunohistochemistry on the SPINOPHILIN protein.

Consequently, low levels of Spinophilin mRNA were also associated with a shorter disease-free interval (DFI) and OS in these series of patients with lung tumors, highlighting the relevance of Spinophilin as a predictive factor (Figure 2 and Supplementary Figure 1).

### Decreased levels of PPP1Cs are related with high risk and predicted poor outcome in lung SCC cancer patients

**Figure 3:**
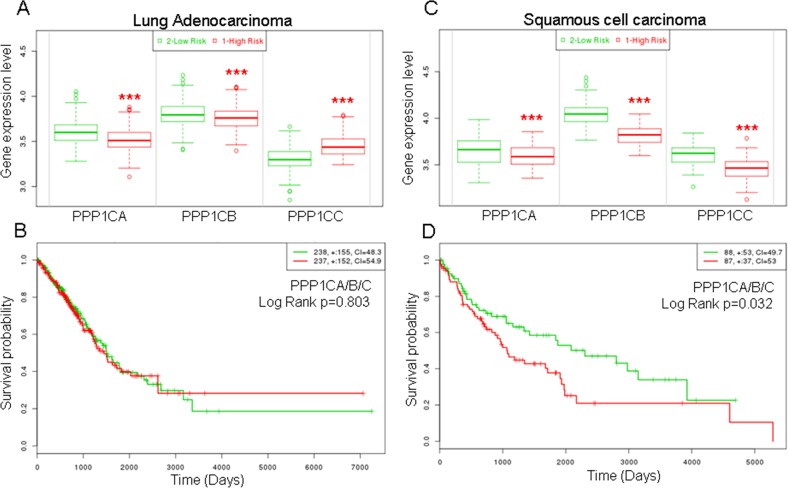
Higher risk and lower survival probability of patients with lung cancer according to lower mRNA levels of the catalytic subunits of PP1 **(A** and **C)** Risk of worse survival probability according to mRNA levels in patients with adenocarcinoma (left, A) or squamous cell carcinoma (right, C). **(B** and **D)** Survival probability (log rank) of patients with lung cancer according to the mRNA levels of the joint catalytic subunits of PP1. Values were taken above or below the average for each subunit evaluated. High or low risks were taken according to the values of figure (A) and (C). (B) Lung adenocarcinoma; (D) Squamous cell carcinoma. The TCGA cohort was used.

SPINOPHILIN is a regulatory subunit of PP1 and binds one of the catalytic subunits, PPP1CA, B or C, forming a heterodimer with PP1 phosphatase activity. Therefore, we measured whether the levels of these catalytic subunits are related to survival probability in tumors of the lung. To this end, we selected the TCGA databases for adenocarcinoma or SCC specific tumors. We observed that for adenocarcinoma, the patients with low levels of PPP1CA or B have a significantly higher risk of decreased survival than patients with high levels of expression of these genes (Figure 3A). The opposite effect is observed for PPP1CC. However, the survival probability of the patients does not change significantly when all three PPPC catalytic subunits are considered (Figure 3B).

When we studied SCC tumors, we observed a more homogeneous behavior and the patients with low levels of PPP1CA, B or C have a significantly higher risk than patients with high levels of these genes (Figure 3C). Furthermore, patients with low levels of these genes have a significantly poorer survival probability (Figure 3D).

**Figure 4:**
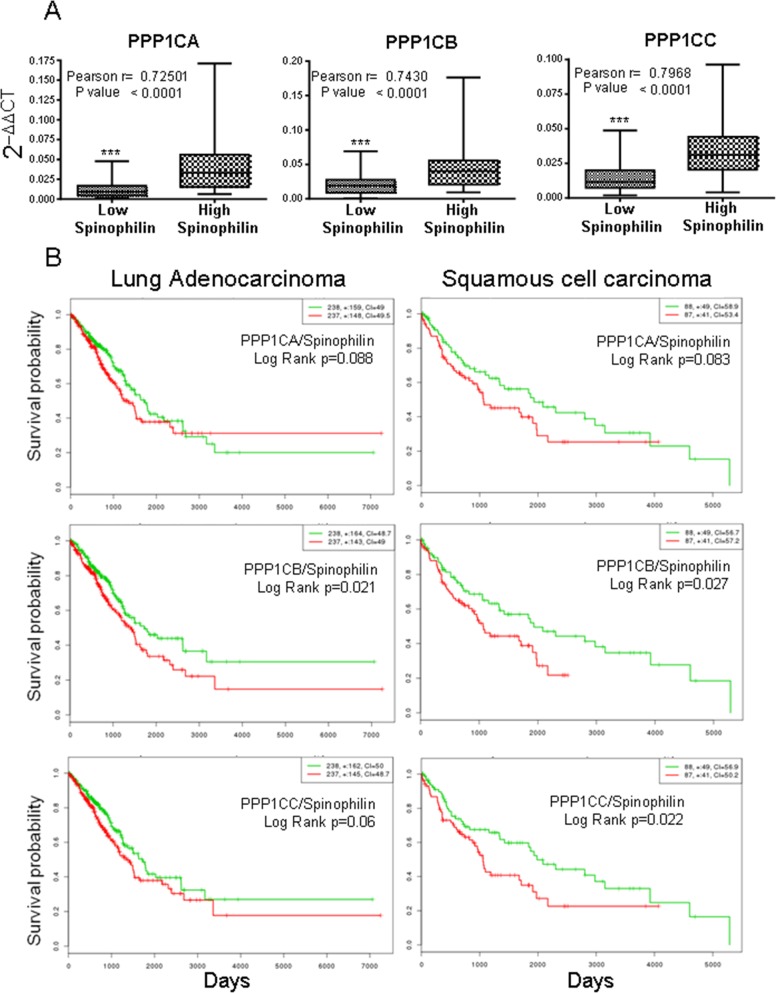
Survival probability of patients with lung cancer according to the joint mRNA levels of the individual catalytic subunits of PP1 and Spinophilin **(A)** Correlation between the mRNA levels of Spinophilin and the catalytic subunits of PP1 (Student's *T*-test; ^***^=p<0.001). We analyzed the levels in a cohort of 70 samples from our cohort from Supplementary Table 1, for which we had mRNA (see Materials and Methods). We quantitated by Q-RT-PCR the levels of PPP1CA, PPP1CB and PPP1CC and plotted according to the low or high levels of Spinophilin mRNA (graph). The graph shows the distribution of the PP1 catalytic subunit according to the categorization of samples in high or low Spinophilin according to its average. Furthermore we plotted one to one correlation of PPP1CA/B/C mRNA levels to Sphinophilin mRNA levels in each sample. The index of correlation (r) and statistical significance (p) for the Pearson correlation is included in the inset in each graph. **(B)** Survival probability (log rank) of patients with lung cancer according to the mRNA levels of the joint individual catalytic subunits of PP1 and Spinophilin. Values were taken above or below the average for each subunit evaluated. High or low risks were taken according to the values of Figure 3A. The TCGA cohort was used.

As a regulatory subunit, we studied the correlation of Spinophilin mRNA expression with those of the different catalytic subunits. We analyzed the levels of PPP1Cs mRNA in samples from Supplementary Table 1. We detected a direct relationship between the levels of Spinophilin and those of each catalytic subunit (Figure 4A, box graph was analyzed by Student's *T*-test; inset also shows Pearson r). Therefore, we next measured whether the combination of regulatory Spinophilin and either of the catalytic subunits might have some predictive capability. We found that in adenocarcinoma tumors, the combination had no clear effect on survival prognosis (Figure 4B). However, the prognosis capability of low Spinophilin combined with low levels of PPP1CA/B or C is clearer in patients with SCC tumors of the lung (Figure 4B).

**Figure 5:**
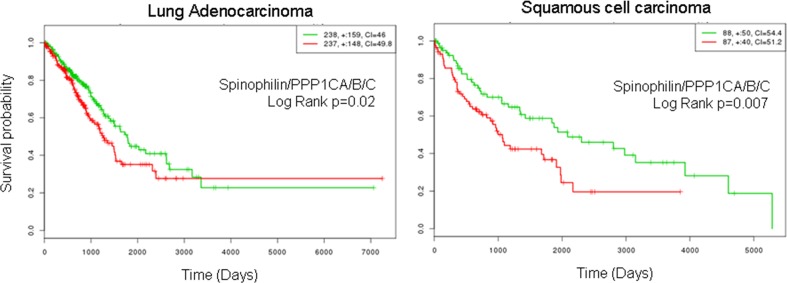
Survival probability of patients with lung cancer according to the joint mRNA levels of the catalytic subunits of PP1 and Spinophilin Survival probability (log rank Cox) of patients with lung cancer according to the mRNA levels of the joint catalytic subunits of PP1 and Spinophilin. Values were taken above or below the average for each subunit evaluated. High or low risks were assessed according to the values of Figure 3A. The TCGA cohort was used.

Finally, we combined low Spinophilin and low PPP1CA/B and C levels and analyzed the predictive capability of survival in patients with adenocarcinoma or SCC tumors. We observed a clear and significant poor prognosis in patients with tumors with low levels of combined Spinophilin/PPP1CA, B and C only in patients with SCC tumors (Figure 5).

While the analysis of the methylation of Spinophilin showed increased methylation, the analysis of PPPCs subunits methylation showed a decreased methylation mean, in tumors vs. non-tumor samples, in PPP1CA and PPP1CB, and increased methylation mean in PPP1CC (See Supplementary Table 4). Therefore, the mechanism of regulation must be different for all three isoforms. It may include transcriptional regulation or cell adaptation throughout the growth of the tumor.

### Analysis of the GO terms correlating to Spinophilin

**Figure 6:**
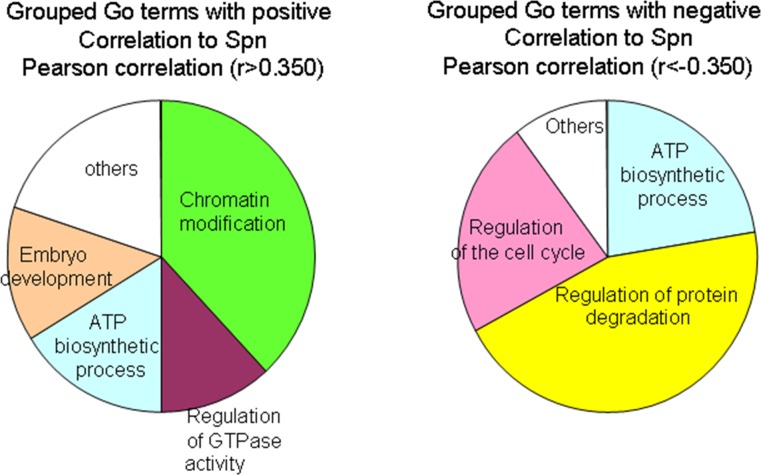
GO term enrichment by genes that correlate positively or negatively to Spinophilin levels The analysis by the Enrichr portal was performed on the genes from Supplementary Table 5. The genes were obtained by R2 analysis in data from the TCGA database.

Patients with lung tumors with low Spinophilin levels showed clear and significant poor prognosis. Therefore, new therapeutic alternatives for these patients are needed. To explore this point, we looked for genes that correlated positively and negatively to Spinophilin (PPP1R9B) in the TCGA database. We selected genes with a correlation r>0.350 or r<-0.350, to identify genes that correlate positively or negatively to Spinophilin levels in tumors (Supplementary Table 5). Next, we identified the GO terms related to these genes using the Enrichr web portal (Supplementary Table 6A and 6B). The GO terms that correlated positively to Spinophilin were enriched in several biological processes, such as chromatin modification, ATP biosynthetic processes, embryo development and the regulation of GTPase activity (Figure 6). Alternatively, GO terms that correlated negatively to Spinophilin, and therefore may be enriched in tumors with low Spinophilin, were the regulation of protein degradation, ATP biosynthetic processes and the regulation of the cell cycle (Figure 6).

These GO term enrichment assays suggest that several pathways interfere with the aim of finding alternative therapies. Processes such as the regulation of protein degradation, ATP biosynthetic processes and the regulation of the cell cycle being negatively correlated seemed more suitable because they are altered in the absence of Spinophilin. Therefore, we tested the effect of metformin, a regulator of the ATP biosynthetic process, and bortezomib, an inhibitor of protein degradation, in a panel of lung cancer cell lines and their correlation to Spinophilin.

### Analysis of the correlation between Spinophilin and PPP1Cs in a panel of lung cancer cell lines and their relationship with the drug response

**Figure 7:**
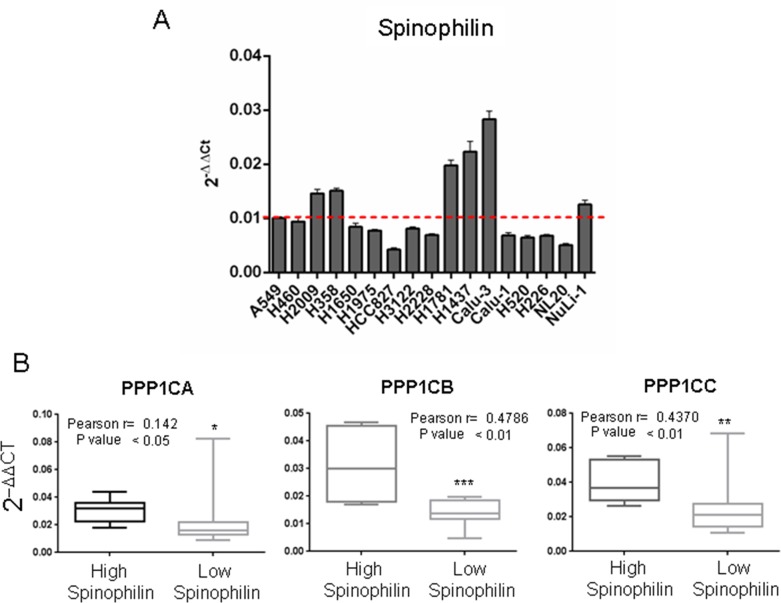
Correlation between the mRNA levels of Spinophilin and the catalytic subunits of PP1 in a panel of tumor cell lines We analyzed a cohort of 17 cell lines described in Supplementary Table 7. **(A)** We analyzed the expression levels of Spinophilin in the cohort of 17 cell lines described in Supplementary Table 3. We divided the panel into high and low Spinophilin, considering high Spinophilin those cell lines with expression > 0.01: H1437, H1781, H2009, H358, Calu3 and Nuli1 cell lines; and low Spinophilin the ones with expression < 0.01: H1650, H1975, H2228, H226, H3122, H460, H520, HCC827, Calu1, A549 and NL20 cell lines. **(B)** We detected a direct relationship between the levels of Spinophilin and the levels of each catalytic subunit. The graph shows the distribution of the PP1 catalytic subunit according to the categorization of samples with high or low Spinophilin according to its average. We considered high Spinophilin: H1431, H1781, H2009, H358, Calu3 and Nuli1 cell lines, and low Spinophilin: H1650, H1975, H2228, H226, H3122, H460, H520, H827, Calu1, A549 and NL20 cell lines. Furthermore, the inset shows Pearson's r and its statistical significance.

First, we analyzed the levels of expression of Spinophilin in a panel of 17 lung cancer cell lines (Supplementary Table 7), and we observed a different pattern of expression (Figure 7A). Therefore, we classified the panel as high Spinophilin cell lines (H1437, H1781, H2009, H358, Calu3 and Nuli1) and low Spinophilin cell lines (H1650, H1975, H2228, H226, H3122, H460, H520, HCC827, Calu1, A549 and NL20).

Then, we studied the correlation of Spinophilin expression with those of the different catalytic subunits in the panel of 17 different lung cancer cell lines (Supplementary Table 8). As in the case of tumors, we detected a direct relationship between the levels of Spinophilin and the levels of each catalytic subunit (Figure 7B, inset shows also Pearson r; Supplementary Table 8). As in tumors, cell lines with low Spinophilin also contained low levels of each catalytic subunit.

With the aim of finding a drug that may be active in lung cell lines with low Spinophilin levels, we subjected this panel of 17 cell lines to different treatments to obtain the IC50 for the response in each cell line (Supplementary Table 9). We specifically tested cisplatin and etoposide as common treatments for lung tumors as well as oxaliplatin as a platinum-derived compound with a different spectrum of activity. Finally, we also tested metformin as a modulator of ATP biosynthesis and bortezomib as an inhibitor of proteasomal degradation [32, 33] because these two mechanisms seemed to be highly related to genes that correlated to Spinophilin levels. Then, we correlated the IC50 for each drug with the levels of Spinophilin, PPP1Cs or the ratio among them and calculated the correlation index (Pearson r). We found that none of the activities of these drugs correlated with the levels of expression of any of the tested genes individually (Table 2). However, we found a clear correlation between the ratio between Spinophilin and PPP1CA or PPP1CB and the activity of oxaliplatin or bortezomib (Table 2, Supplementary Figure 2). Alternatively, we did not observe a correlation between metformin activity and Spinophilin or PP1 values.

**Table 2:** Pearson correlation (r) between the IC50 of the different treatments and the levels of the indicated genes

	Spinophilin	PPP1CA	PPP1CB	PPP1CC	Spinophilin/PPP1CA^*^	Spinophilin/PPP1CB^*^	Spinophilin/PPP1CC^*^
**metformin**	−0.2567736	−0.0305505	0.0471795	0.0072612	−0.1737827	−0.2997706	−0.3934922
**oxaliplatin**	0.1098795	−0.0758366	−0.2116024	0.2138328	**0.5907283**	**0.6886702**	0.0679746
**cisplatin**	0.0367228	0.06338384	0.0771211	−0.1303417	−0.1979426	−0.1891476	−0.0018704
**etoposide**	0.0134608	−0.0350167	−0.0008127	−0.0196913	−0.0707570	−0.0349190	−0.0275979
**bortezomib**	−0.0652384	−0.3027230	−0.0553075	0.2275598	**0.7996544**	**0.4329986**	0.0972166

^*^ indicates the ratio between the levels of Spinophilin and the catalytic subunit indicated.

These data suggest that the lower ratio between the levels of expression of these genes may be a good marker for the response to oxaliplatin or bortezomib. Therefore, tumors with lower levels of Spinophilin might respond better to oxaliplatin and/or bortezomib depending on the levels of PPP1CA/B. However, this functional hypothesis needs more research and to be validated in animal models.

## DISCUSSION

Downregulation of Spinophilin, either in protein or mRNA, is related to worse prognosis in lung tumors. This effect is more relevant in squamous cell carcinoma than in adenocarcinoma. Downregulation of Spinophilin is related to a decrease in the levels of PPP1CA/B/C, the catalytic subunits of PP1 and partners of Spinophilin. A decrease in these subunits is also related to a poor prognosis in SCC and is observed more clearly in combination with a decrease in Spinophilin. PP1 has been identified as the major enzyme that dephosphorylates pRb during mitosis [34, 35] and plays an important role in the G1/S transition [36]. Although there is literature supporting the role of PP1 regulation of pRb *in vivo* [37–39], our work is the first to support the downregulation of the components of the PP1 heterodimer as a direct contribution to lung cancer and as a predictor of a worse prognosis for these patients.

Along with lung tumors [7], Spinophilin mRNA and/or proteins are lost in a percentage of different neoplasias [23]. Spinophilin levels were also associated with high proliferative recurrences and poor patient prognosis in hepatocarcinoma, head and neck cancer and advanced stages of colorectal carcinoma [8–11]. Furthermore, lower levels of Spinophilin mRNA correlated with a higher grade of renal carcinomas, ovarian carcinoma and chronic myelogenous leukemia [23]. In human breast tumors, Spinophilin is lost or reduced in approximately 15% of samples, correlating with higher grade and more aggressive neoplasms [14]. Spinophilin loss correlates with a higher level of a putative CSC-like phenotype [11, 14].

Spinophilin regulates PP1 activity [40], and the loss of Spinophilin reduces the phosphatase activity of PP1a on its target pRb [41], thereby maintaining higher levels of phosphorylated pRb and inducing an increased proliferative response [21]. The pRb pathway controls several aspects of stem cell biology, including the tight control of self-renewal characteristics of progenitor cells [42, 43]. It has been suggested that relapse and poor response to chemotherapy is related to the number of CSCs [44]. The fact that the loss of Spinophilin increases the stem-like properties of tumor cells may explain its association with more aggressive tumors and poor response in patients.

It is remarkable that PPP1CA downregulation mimics the effect of Spinophilin downregulation, increasing the proportion of cancer-initiating cells, suggesting that cells with low PP1 phosphatase activity are characterized by aggressive features and may encompass a higher percentage of precursors than the putatively less aggressive counterparts [44]. These results fully support those of Dedinszki et al., who showed that inhibition of protein phosphatase-1a decreases the chemosensitivity of leukemic cells to chemotherapeutic drugs [45].

The loss of pluripotency and stemness is associated with the activation of pRb, which determines the transcription of E2F-dependent genes [42]. Because pRb activity is directly regulated by the PP1 heterodimer studied in this work, there is the possibility that some of the clinical properties observed *in vivo* may be due to the pRb activity regulating cancer stemness [43]. Additionally, consistent with our results, Spinophilin can restrain the self-renewal of brain tumor-initiating cells [46] and anchorage-independent growth of glioma cell lines [47]. In addition to the PP1 regulatory activity of Spinophilin on pRb phosphorylation, Spinophilin has other targets, such as doublecortin, an actin-binding protein with an established role in the subcellular targeting of PP1 [17, 48]. Spinophilin enhances PP1-mediated dephosphorylation of the PSer297 site of doublecortin [20]. Doublecortin is a microtubule-binding protein that induces growth arrest at the G2–M phase of the cell cycle in glioma cells and suppresses tumor xenograft growth in a Spinophilin-dependent manner, which occurs concomitantly with PP1 localization into the cytosol [47]. Doublecortin significantly reduces self-renewal of brain tumor stem cells in human primary glioma cells from surgically removed human glioma specimens and glioma cells *in vitro* and *in vivo* [46]. This effect on the restriction of self-renewal of brain tumor-initiating cells appears dependent on Spinophilin expression [46]. To what extent there are two independent effects, on pRb and doublecortin or additive or synergistic effects, should be further studied.

According to the expression data provided by the Project: HPA RNA-seq normal tissues where RNA-seq was performed of tissue samples from 95 human individuals representing 27 different tissues in order to determine tissue-specificity of all protein-coding genes [49]. All three isoforms are ubiquitously expressed, but with some variation in the levels according to the different tissue analyzed. PPP1CA mRNA is mainly found in cells of the gastro-intestinal organs, bone marrow, spleen, lymph node and bladder; PPP1CB mRNA is mainly found in cells from prostate, heart and endometrium. PPP1CC mRNA is mainly found in cells from the gastro-intestinal organs and testis. The catalytic subunits may be functionally equivalent biochemically, however, since the specificity on the effector is given by the regulatory proteins, the later are the ones conferring different functionality to PP1 heterodimers on cells.

While the analysis of the methylation of Spinophilin showed increased methylation, the analysis of PPPCs subunits methylation showed a decreased methylation mean, in tumors vs. non-tumor samples, in PPP1CA and PPP1CB, and increased methylation mean in PPP1CC. Therefore, the mechanism of regulation must be different for all three isoforms. Combined to the data of Spinophilin, it indicates that the regulation of the heterodimer of PP1 is not homogeneous by methylation. These data further suggests that the combined co-regulation of PPPCs and the regulatory subunit Sphinophilin may include strong transcriptional regulation or cell adaptation throughout the growth of the tumor. This is an interesting point that should be further explored.

Because phosphatase regulators exist in a molar excess to PPP1Cs [50], how the downregulation of one single regulator protein, even partial, such as Spinophilin, triggers such important regulatory effects leading to cancer is unknown. We can argue that it is the specific effector of PP1 targeted by the regulator that is truly the key point of the effect. Alternatively, PPP1Cs recruitment is associated with the folding of the regulators, and it can be hypothesized that some PP1 functions may be independent of its enzymatic activity and involve some type of chaperone function [50].

Other regulators targeting similar components, such as PPP1R12a (MYPT1), are also downregulated in tumors [51, 52] but have no effect on patient survival with lung tumors nor alter the effect of the partner catalytic subunits (data not shown).

Finally, in a panel of lung cancer cell lines, the analysis of the response to several commonly used drugs indicates a direct correlation between the Spinophilin/PPP1C ratio and the response to oxaliplatin or bortezomib. This finding indicates that this ratio may be a good marker for the activity of these drugs in tumors with a poor prognosis. Spinophilin-correlating genes and GO-related networks suggest a relationship to the ubiquitin degradation pathway with Spinophilin, which may explain this effect. However, although we found some relationship to DNA repair components, it is difficult to differentiate the positive effect of oxaliplatin from that not responding to cisplatin or etoposide. It will also be interesting to test the relationship of Spinophilin levels to cell cycle inhibitors or chromatin modifiers also according to the results of the GO enrichments.

In summary, our data show for the first time that the protein Spinophilin has prognostic and predictive value for lung cancer. Interestingly, we observed certain co-regulation between Spinophilin and the catalytic subunits of PP1. The low levels of these subunits also have prognostic and predictive value, especially in squamous cell lung carcinoma, and the combination of low levels of Spinophilin+PPP1Cs has stronger prognostic value. Finally, in a panel of lung cancer cell lines, the analysis of the response to several commonly used drugs indicates a direct correlation between the Spinophilin/PPP1C ratio and the response to oxaliplatin or bortezomib. This finding indicates that this ratio may be a good marker for the activity of these drugs in these tumors with poor prognosis.

## MATERIALS AND METHODS

### Human samples

The study was performed in 2 different cohorts.

The first cohort comprises a group of 245 NSCLC patients from Virgen del Rocío Hospital. All samples were collected from 2007–2009. This cohort is reported in Supplementary Table 1. No patients were treated with chemotherapy or radiotherapy before surgical resection. Written informed consent was obtained from all of the patients, and the Ethical Committee of the Hospital involved approved the study. All of the samples were treated according to the Helsinki guidelines for research regarding human samples. From this cohort we generated a tissue microarray that was stained for Spinophilin and p53 protein levels. These data was used in Figures 1A and 2. From this cohort, we could obtain mRNA from a subgroup of 72 patients only. The mRNA from this subgroup was only used for the quantitative experiments of Figures 1B and 4A.

The second cohort, described in the Supplementary Table 2, contains 70 DNA samples from, 47 patients not included in the first cohort. It was composed of 23 matched tumor and non-tumor samples from the same patient, plus 24 non-matched tumor samples from different patients. In total, only 47 patients composed the second cohort. The clinical features of patients with NSCLC of this cohort are summarized in Supplementary Table 2. The samples were obtained from patients following surgical resection mostly for clinical early stage NSCLC, but also included 10 samples from stages III and IV. A description of this cohort can also be found in the literature [53]. During the surgical procedure, the tumor and matched non-tumor tissue samples were collected from patients and then immediately snap-frozen at −80°C for future use. The methylation profiles of Spinophilin and PPCs were evaluated in tumoral and non-tumoral tissue. A written consent form was obtained from all participants. The study protocol and the use of human samples were approved by the Ethical Committee of the Virgen del Rocio University Hospital.

### DNA samples

Genomic DNA was extracted from tumor and matched non-tumor tissue samples by the QIAamp DNA mini kit (QIAGEN, Valencia, CA, USA). DNA was quantified using the QuantiFluor dsDNA system (Promega, Madison, WI, USA) according to the manufacturers’ instructions.

### Illumina 450 K methylation

The Illumina Infinium Human Methylation 450 BeadChip (Illumina Inc., San Diego, CA, USA) was used to interrogate 485,000 methylation sites across the genome per sample at single-nucleotide resolution. It covers 96% of the CpG islands, with additional coverage in island shores and the flanking regions. We treated 500 ng of DNA with sodium bisulfate using the EZ DNA Methylation™ Kit and cleaned the DNA with the ZR-96 DNA Clean-up Kit™ (EZ DNA, Zymo Research, Irvine, CA, USA) before standard Illumina amplification, hybridization, and imaging steps. The resulting intensity files were analyzed with Illumina's GenomeStudio, which generated β-scores (i.e., the proportion of total signal from the methylation-specific probe or color channel).

### Methylome data processing

Methylome data were processed using the RnBeads R package [54]. After a quality check, the probe median intensity was normalized with the SWAN method [55] and converted to beta values. The probes were tested for differential methylation with the limma method, a linear model followed by empirical Bayes methods for the comparisons of interest [56]. The CpG status (hypo- versus hyper-methylated) and CpG chromosomal location were realized using the Circos data visualization software [57].

### Analysis of gene transcription

Total RNA was purified using an RNeasy Kit (QIAGEN) and reverse transcription into cDNA was performed with 3 μg of mRNA using the High-Capacity cDNA Reverse Transcription Kit (Life Technologies) according to the manufacturer's recommendations. Real-time PCR was performed with an Applied Biosystems 7900HT cycler using the GoTaq® Probe qPCR Master Mix (Promega), following the manufacturer's recommendations. The thermocycler parameters were 95°C for 10 min followed by 40 cycles of 95°C for 15 s and 60°C for 60 s. We used the following TaqMan gene-specific probes from Life Technologies: *PPP1R9B* (Hs00261636_m1), *PPP1CA*(Hs00267568_m1), *PPP1CB* (Hs01027793_m1), *PPP1CC* (Hs00160351_m1), and *GAPDH* (Hs03929097_g1). We used the housekeeping gene *GAPDH* to normalize the RNA amount. Relative changes in gene expression levels were calculated using the comparative threshold cycle (ΔΔCt) method. At least three independent experiments were performed for each of the analyzed genes. Student's *t*-test was applied for each pair of samples, with a significance threshold of p < 0.05.

### Immunohistochemistry

Immunohistochemistry analysis was performed as previously described [58–60]. The primary antibody (Anti-Spinophilin: ab5669 from Merck Millipore) was incubated overnight at 4°C as previously described [58, 61, 62]. A secondary anti-rabbit antibody (JI-111-035-003) was applied for 1 hour at room temperature and revealed using substrate buffer and chromogen (Envision, Flex DAKO). The tissues were counterstained with hematoxylin (DAKO), rehydrated in a graded alcohol series, and mounted using coverslips. These procedures were performed at the Histopathology Unit at the IBIS.

### Retrospective analysis of gene expression in human tumors

Correlations between grade, patient survival, tumor recurrence and Spinophilin and PPP1CA/B/C gene expression were determined through analysis of French (GSE16011), TGCA, French-Core Exon (GSE43107), Sun Brain (GSE4290) and Freije (GSE4412) datasets, respectively, which are available through Oncomine (Compendia Biosciences,www.oncomine.org) and R2: Genomics analysis and visualization platform (http://r2.amc.nl/). High and low groups were defined as above and below the mean, respectively. To analyze the high and low groups, high was defined as greater than one standard deviation above the mean, and low was greater than one standard deviation below the mean. The National Cancer Institute's Repository for Molecular Brain Neoplasia Data (REMBRANDT,http://rembrandt.nci.nih.gov) was also evaluated for correlations between patient survival and gene expression with up- or downregulation being defined as a 2-fold change relative to mean values. Multigene analysis of Kaplan-Meier (Cox regression) curves was performed through the *SurvExpress* Genomics analysis and visualization platform [63].

### Statistical analysis

All grouped data are presented as the mean ± standard error. The difference between groups was assessed by ANOVA or Student's *t*-test using GraphPad Prism software. For survival analysis, Kaplan-Meier curves were generated using Prism software and R2 Kaplan-Meier plotting service and log rank analysis was performed. All experiments were repeated in each condition at least twice with triplicate technical replicates. The data distribution was assumed to be normal but was not formally tested. Data obtained for retrospective analysis were collected and processed in the appropriate experimental arms.

Multivariate analysis was performed with the Cox proportional hazards method. In these analyses, overall survival (OS) and progression free survival (PFS) were defined as the time from diagnosis to exitus and progression, respectively. P<0.05 was considered significant. All analyses were performed using the Statistical Package for the Social Sciences software (SPSS 17.0 for Windows; SPSS Inc, Chicago, IL, USA).

### Cell lines

Two immortalized lung epithelial cell lines (NuLi-1 and NL-20), 4 lung SCC cell lines (Calu-1, HTB59, H520 and H226) and 12 lung ADC cell lines were used (Supplementary Table 3). All cell lines were purchased from the ATCC before the beginning of this work, with the exception of H1437 and H3122, which were kindly provided by Dr. Maina and Dr. Koivunen, respectively. No further authentication was performed in these cell lines. All cell lines were culture in RPMI 1640 medium (Sigma), except for Calu-1, which was cultured in McCoy's 5A medium (Gibco) and Calu-3 in DMEM medium (Sigma), supplemented with 10% fetal bovine serum (FBS) at 37°C under a 5% CO2 atmosphere. All cell lines were regularly tested for mycoplasma.

### MTT assay

For the assay, 5×10^3^ cells were seeded and then treated with the different compounds (oxaliplatin, cisplatin, etoposide, metformin and bortezomib) at 11 different concentrations at 1/3 after 24 hours. Then, 96 hours later, cell viability was measured via MTT assay.

## SUPPLEMENTARY MATERIALS FIGURES AND TABLES








