# Supplementary material for: Coordinated downregulation of Spinophilin and the catalytic subunits of PP1, PPP1CA/B/C, contributes to a worse prognosis in lung cancer
Source: Oncotarget. 2017 Oct 26;8(62):105196–210. doi: 10.18632/oncotarget.22111 (PMC5739631; doi:10.18632/oncotarget.22111)
Supplement: Supplementary file 1 [file oncotarget-08-105196-s001.pdf]

Coordinated downregulation of Spinophilin and the catalytic subunits of PP1, PPP1CA/B/C, contributes to a worse prognosis in lung cancer

SUPPLEMENTARY MATERIALS

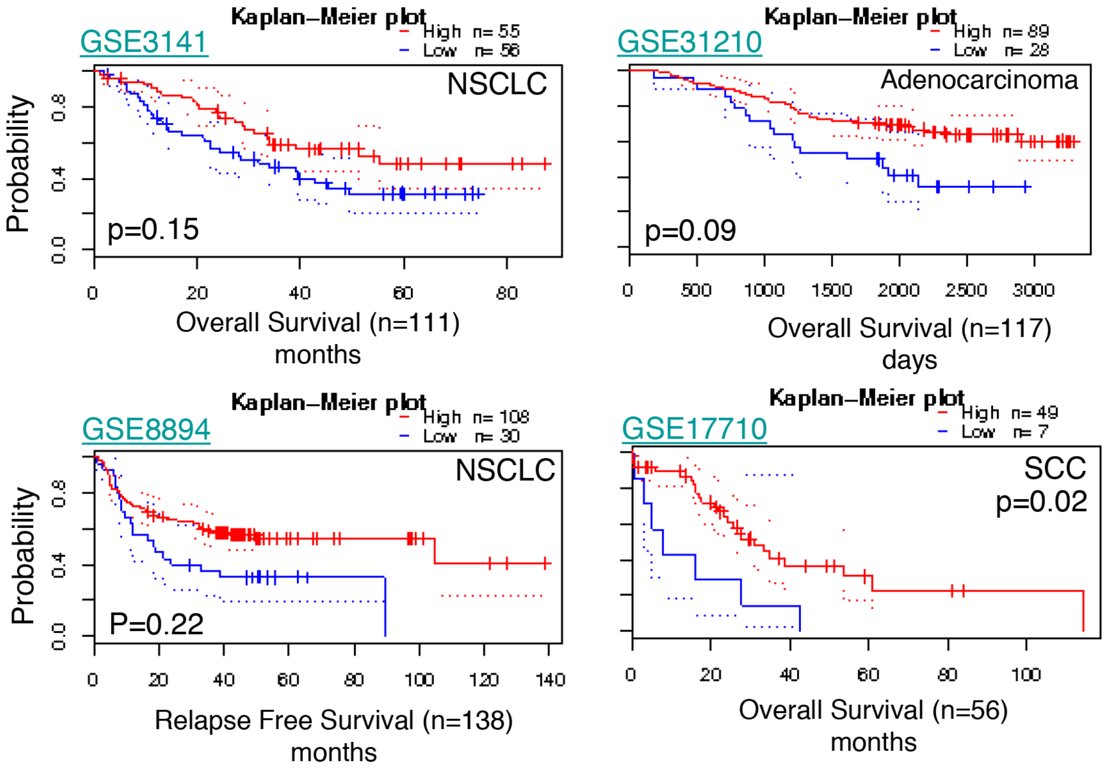

Supplementary Figure 1: Survival probability of patients with lung cancer according to Spinophilin levels in different lung cancer patient cohorts as indicated.

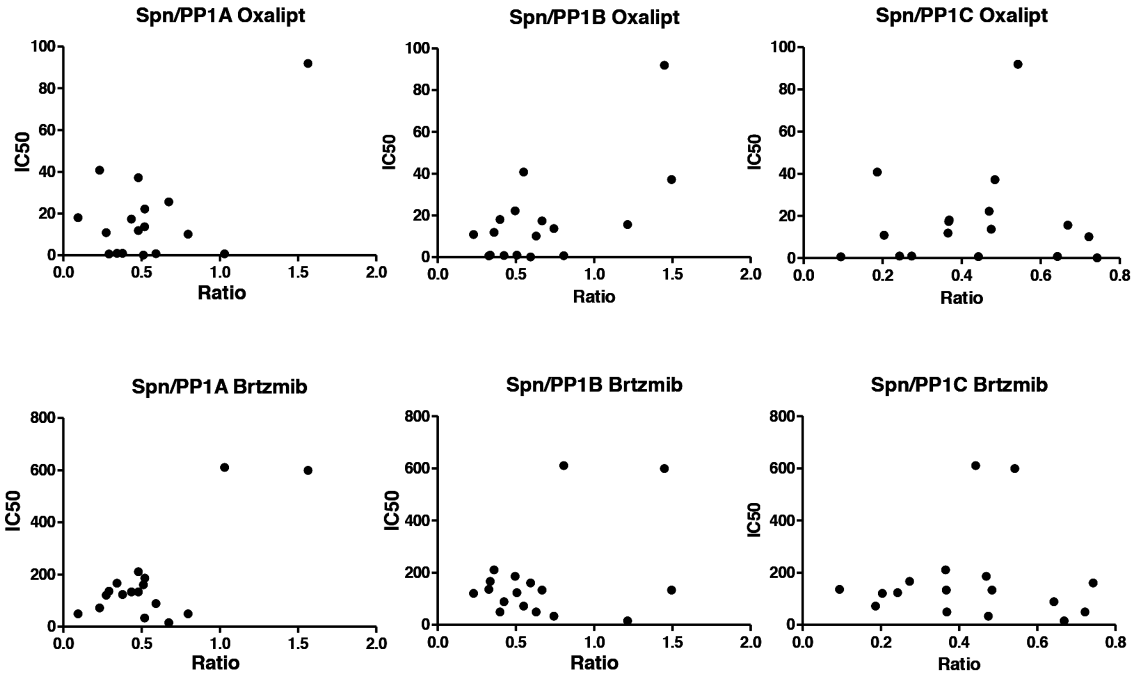

Supplementary Figure 2: Graphs of the correlation between Oxaliplatin (Oxlipt) or Bortezomib (Brtzmib) and the ratios Spinophilin/PPP1CA (Spn/PP1A), Spinophilin/PPP1CB (Spn/PP1B), Spinophilin/PPP1CC (Spn/PP1C). (Pearson correlation).

Supplementary Table 1: Clinicopathological characteristics of the NSCLC cohort number 1

See Supplementary File 1

Supplementary Table 2: Characteristics of the lung cancer patient cohort used in the methylation assay. Cohort number 2

	Patients (N=47)
**Gender**	
Male	76.6 (36)
Female	23.4 (11)
**Age (years)**	67 [60–73]
**Smoking status**	
Smokers	40.4 (19)
Ex-smokers	44.7 (21)
Non-smoker	14.9 (7)
**Packs-year**	41.0 [20.0-65.7]
**Histology**	
Lung adenocarcinoma	57.4 (27)
Squamous cell carcinoma	42.6 (20)
**Staging**	
Stage I	40.5 (19)
Stage II	38.3 (18)
Stage III-IV	21.2 (10)
**Subjects with COPD**	42.6 (20)

Continuous variables are expressed as median [interquartile range (IQR)] and categorical variables as number of cases (%).

Supplementary Table 3: Multivariate analysis for PFS and OS in lung cancer patients

Variable	PFS		OS	
	HR (IC 95%)	p-value	HR (IC 95%)	p-value
Age	1.013 [0.991-1.035]	0.246	0.998 [0.352-2.200]	0.895
Sex	0.753 [0.309-1.835]	0.533	0.960 [0.387-2.384]	0.931
ECOG	1.680 [1.089-2.593]	0.019	1.437 [922-2.241]	0.110
Stage	1.269 [1.086-1.483]	0.003	1.212 [1.036-1.418]	0.016
Smoking habits	1.366 [0.924-2.019]	0.118	1.190 [0.799-1.774]	0.392
SPINOPHILIN	1.683 [1.092-2.596]	0.020	1.699 [1.192-2.446]	0.022

Supplementary Table 4: Methylation of PPP1CA/B and C promoters in lung tumors

Smokers	No cancer	Cancer	p
PPP1CA	0.3168	0.2506	0.000016
PPP1CB	0.2906	0.1538	0.032025R2
PPP1CC	0.4762	0.5516	0.000007
Adc (smokers)	NO CANCER	CANCER	p
PPP1CA	0.3156	0.2616	0.011022
PPP1CB	0.2863	0.1563	0.000011
PPP1CC	0.4727	0.5399	0.005708
SCC (Smokers)	NO CANCER	CANCER	p
PPP1CA	0.3237	0.2395	0.0024789
PPP1CB	0.3053	0.1512	0.032025R21
PPP1CC	0.4794	0.5634	0.0026520
Non-Smokers	NO CANCER	CANCER	p
PPP1CA	0.2925	0.2500	0.338367
PPP1CB	0.2840	0.2568	0.499554
PPP1CC	0.4529	0.4702	0.628074

The data shows the average mean of methylation (see Methods) in samples analyzed. In all cases Student's T test was performed and the significance indicated (p).

Supplementary Table 5: List of genes that correlated positively or negatively with Spn (PPP1R9B), Pearson correlation r>0,350 or r<-350 (p<0.05) in the Lung Squamous cell carcinoma TCGA database (n=81). Correlation was performed in R2: Genomics Analysis and Visualization Platform. Supplementary Table 3 is provided in the excel document.

See Supplementary File 2

Supplementary Table 6A: GO biological processes affected by the genes with positive correlation to Spinophilin in Squamous cell lung tumors (TCGS database). Based on Gene list from Supplementary Table 3. Performed through Enrichr web page (http://amp.pharm.mssm.edu/Enrichr/enrich)

See Supplementary File 3

Supplementary Table 6B: GO biological processes affected by the genes with negative correlation to Spinophilin in Squamous cell lung tumors (TCGS database). Based on Gene list from Supplementary Table 3. Performed through Enrichr web page (http://amp.pharm.mssm.edu/Enrichr/enrich).

See Supplementary File 3

Supplementary Table 7: Summary of the different cell lines used in our study

Cell line	Histology	Driver mutation	Reference for driver mutation
**A549**	ADC	KRAS p.G12S	(Helfrich, Raben et al. 2006)
**H460**	ADC	KRAS p.Q61H	(Helfrich, Raben et al. 2006)
**H2009**	ADC	KRAS p.G12A	COSMIC
**H358**	ADC	KRAS p.G12C	(Helfrich, Raben et al. 2006)
**H1650**	ADC	EGFR E746-E750 del	(Blanco, Iwakawa et al. 2009)
**H1975**	ADC	EGFR L858R/T790M	COSMIC
**HCC827**	ADC	EGFR E746-E750 del	(Helfrich, Raben et al. 2006)
**H3122**	ADC	EML4-ALK v1 translocation	COSMIC
**H2228**	ADC	EML4-ALK v3 translocation	COSMIC
**H1781**	ADC	TN (*L858R mutation detected in our lab)	(Helfrich, Raben et al. 2006)
**H1437**	ADC	TN	(Helfrich, Raben et al. 2006)
**Calu-3**	ADC	TN	(Helfrich, Raben et al. 2006)
**Calu-1**	SSC	KRAS p.G12C	COSMIC
**HTB59**	SSC	KRAS p-G12V	COSMIC
**H520**	SSC	TN	COSMIC, (Helfrich, Raben et al. 2006)
**H226**	SSC	TN	COSMIC, (Helfrich, Raben et al. 2006)
**NL20**	NT	TN	COSMIC
**NuLi-1**	NT	TN	COSMIC

ADC=Adenocarcinoma, SCC=Squamous Cell Carcinoma, TN=Triple Negative, NT=Non-Tumorogenic.

Supplementary Table 8: Levels of mRNA expresión of different subunits of PP1

	Spinophilin		PPP1CA		PPP1CB		PPP1CC	
	Average	StDev	Average	StDev	Average	StDev	Average	StDev
1431	0.0223	0.0018	0.0331	0.0031	0.0184	0.0013	0.0334	0.0029
1650	0.0084	0.0006	0.0105	0.0006	0.0134	0.0007	0.0116	0.0011
1781	0.0197	0.0009	0.0334	0.001	0.0467	0.0023	0.0307	0.001
1975	0.0077	0.0002	0.0822	0.0036	0.0194	0.0014	0.021	0.0013
2009	0.0146	0.0007	0.0304	0.0013	0.0405	0.0021	0.0399	0.0028
2228	0.0069	0.0002	0.0144	0.0007	0.0046	0.0001	0.0143	0.0008
226	0.0067	0.0002	0.0179	0.0007	0.0134	0.0007	0.0279	0.0011
3122	0.0081	0.0003	0.0158	0.0004	0.0136	0.0015	0.0109	0.0002
358	0.0151	0.0004	0.044	0.0012	0.0449	0.0025	0.0552	0.0043
460	0.0094	0.0009	0.0091	0.0009	0.0116	0.0012	0.0212	0.0026
520	0.0064	0.0004	0.022	0.0013	0.0197	0.0007	0.0683	0.0039
827	0.0042	0.0003	0.0155	0.0013	0.0185	0.0014	0.0208	0.0017
Calu1	0.0068	0.0004	0.0132	0.0011	0.0139	0.0008	0.0146	0.0009
Calu3	0.0283	0.0014	0.0181	0.001	0.0195	0.0008	0.0522	0.0028
A549	0.01	0.0001	0.0232	0.0009	0.0151	0.0008	0.0274	0.0008
NL20	0.005	0.0003	0.0217	0.001	0.0091	0.0006	0.027	0.0011
Nuli1	0.0125	0.0008	0.0242	0.0012	0.0169	0.0009	0.0265	0.0021

High Spn: 1431, 1781, 2009, 358, Calu3, Nuli1.
Low Spn: 1650, 1975, 2228, 226, 3122, 460, 520, 827, Calu1, A549, NL20.

Supplementary Table 9: IC50 of different drugs tested in the panel of lung cancer cell lines

	Oxaliplatin		Cisplatin		etoposide		Metformin		Bortezomib	
	IC50 (uM)		IC50 (uM)		IC50 (uM)		IC50 (mM)		IC50 (uM)	
	Average	StDev	Average	StDev	Average	StDev	Average	StDev	Average	StDev
**A549**	1.7413	0.0280	10.339	4.1085	4.0363	5.5257	3.2386	1.1515	13.3701	1.0221
**Calu-1**	2.2225	1.0236	0.2142	0.0665	1.1534	1.0104	11.5545	2.5617	18.6490	5.2137
**Calu-3**	9.1953	1.1379	0.4032	0.2184	3.5788	4.8779	15.1990	6.7110	59.9610	6.7221
**H1437**	2.5605	0.1175	4.3899	0.1922	2.3559	2.1435	7.0823	1.5668	15.654	0.5861
**H1650**	1.0148	0.2565	6.1025	2.5515	2.4579	3.1789	10.7731	1.6111	4.9663	0.8926
**H1781**	0.7617	0.1123	0.0638	0.0235	0.3126	0.3896	8.30844	2.3046	8.9041	0.3519
**H1975**	1.8063	0.3677	5.3548	0.5610	2.5096	2.5668	15.6023	1.2434	4.9767	0.6480
**H2009**	1.1878	0.3026	15.2050	2.5136	5.5651	8.3600	14.5725	0.2609	21.0881	4.7322
**H2228**	3.7237	0.1539	3.6015	0.2659	2.4931	2.0266	12.7885	4.1366	13.3823	4.9259
**H226**	0.9970	0.1514	4.9107	0.1462	2.0197	2.5391	12.4089	3.5138	12.3963	0.6455
**H3122**	0.0852	0.0109	6.3104	0.8980	2.1355	3.6157	8.8271	0.3994	16.0860	7.2781
**H358**	0.9965	0.3338	0.1946	0.0856	0.5083	0.4285	14.4783	1.1773	16.7320	0.3541
**H460**	0.7449	0.1413	1.9565	0.5650	0.9476	0.9244	10.3559	1.8699	61.1045	1.5916
**H520**	0.5943	0.1953	2.6998	1.0764	1.1631	1.3456	14.2986	1.6980	13.6138	2.7517
**HCC827**	1.0856	0.4212	6.0309	0.9970	2.5126	3.0650	48.1803	15.7150	12.0709	0.5697
**HTB59**	12.3497	3.5491	34.1221	6.1334	16.6736	15.7384	0.4623	0.1653	7.1309	1.0561
**NL20**	4.0820	1.0444	0.0453	0.0059	1.7239	2.1023	9.4784	0.6953	7.2196	0.3583
**NuLi-1**	1.3710	0.4178	0.0370	0.0083	0.6086	0.687	4.0813	1.8089	3.3829	0.7317

Data from 3 independent experiments performed in triplicate.
