# Supplementary material for: Coordinated downregulation of Spinophilin and the catalytic subunits of PP1, PPP1CA/B/C, contributes to a worse prognosis in lung cancer
Source: Oncotarget. 2017 Oct 26;8(62):105196–210. doi: 10.18632/oncotarget.22111 (PMC5739631; doi:10.18632/oncotarget.22111)
Supplement: Supplementary file 2 [file oncotarget-08-105196-s002.docx]

**Supplementary Table 1: Clinicopathological characteristics of the NSCLC cohort number 1**

| **Variable** | **NSCLC (N=248)** |
| --- | --- |
| **Gender**  **Male**  **Female** | 233(94.0%)  15(6.0%) |
| **ECOG Performance status**  **0**  **1**  **2**  **Unknown** | 167(67.3%)  72(29.0%)  3(1.2%)  6(2.4%) |
| **Age** | 66 [39-84] |
| **Smoking habits**  **Ex smoker**  **Current smoker**  **Never smoker**  **Unknown** | 113(45.6%)  120(48.4%)  11 (4.4%)  4(1.6%) |
| **Histology**  **Squamous cell carcinoma**  **Adenocarcinoma**  **Large cell carcinoma**  **Other** | 123 (49.6%)  77 (31.0%)  22 (8.9%)  26 (10.4%) |
| **Stage**  **IA**  **IB**  **IIA**  **IIB**  **IIIA**  **Unknown** | 27 (10.9%)  114 (46.0%)  4 (1.6%)  65 (26.2%)  37 (14.9%)  1 (0.4%) |
| **Tumour differentiation**  **Well differentiated**  **Moderately differentiated**  **Poorly differentiated**  **Unkown** | 18 (7.3%)  78 (31.5%)  98 (39.4%)  54 (21.8%) |
| **Adjuvant radiotherapy**  **Yes**  **No**  **Unknown** | 30 (12.1%)  205 (82.7%)  13 (5.2%) |
| **Adjuvant chemotherapy**  **Yes**  **No**  **Unknown** | 24 (9.7%)  213 (85.9%)  11 (4.4%) |
| **Relapse**  **Yes**  **No**  **Unknown** | 119 (48.0%)  109 (44.0%)  20 (8.1%) |
| **Exitus**  **Yes**  **No**  **Unknown** | 153 (61.7%)  76 (30.6%)  19 (7.7%) |
| **Reason for Exitus**  **Progression**  **Not related**  **Undetermined**  **Surgical complications**  **Toxicity**  **Second tumour** | 104 (41.9%)  22 (8.9%)  107 (43.1%)  4 (1.6%)  10 (4.0%)  1 (0.4%) |

Continuous variables are expressed as median [interquartile range] and categorical variables are expressed as the number of cases (percentage).
