# Supplementary material for: Coordinated downregulation of Spinophilin and the catalytic subunits of PP1, PPP1CA/B/C, contributes to a worse prognosis in lung cancer
Source: Oncotarget. 2017 Oct 26;8(62):105196–210. doi: 10.18632/oncotarget.22111 (PMC5739631; doi:10.18632/oncotarget.22111)
Supplement: Supplementary file 4 [file oncotarget-08-105196-s004.docx]

**Supplementary Table 6A:** **GO biological processes affected by the genes with positive correlation to Spinophilin in Squamous cell lung tumors (TCGS database). Based on Gene list from Supplementary Table 3. Performed through Enrichr web page (http://amp.pharm.mssm.edu/Enrichr/enrich)**

| **Index** | **Name** | **Adjusted p-value** | **Z-score** |
| --- | --- | --- | --- |
| 1 | chromatin modification (GO:0016568) | 1,10E-06 | -2.40 |
| 2 | histone modification (GO:0016570) | 3,51E-06 | -2.40 |
| 3 | covalent chromatin modification (GO:0016569) | 3,51E-06 | -2.40 |
| 4 | positive regulation of GTPase activity (GO:0043547) | 0.0005394 | -2.40 |
| 5 | positive regulation of GTP catabolic process (GO:0033126) | 0.0005394 | -2.40 |
| 6 | histone lysine methylation (GO:0034968) | 0.0003311 | -2.19 |
| 7 | peptidyl-lysine modification (GO:0018205) | 0.0005874 | -2.25 |
| 8 | histone acetylation (GO:0016573) | 0.0007672 | -2.14 |
| 9 | histone methylation (GO:0016571) | 0.001211 | -2.22 |
| 10 | internal peptidyl-lysine acetylation (GO:0018393) | 0.001140 | -2.15 |
| 11 | peptidyl-lysine acetylation (GO:0018394) | 0.001211 | -2.16 |
| 12 | internal protein amino acid acetylation (GO:0006475) | 0.001388 | -2.15 |
| 13 | protein acylation (GO:0043543) | 0.001703 | -2.20 |
| 14 | protein acetylation (GO:0006473) | 0.003148 | -2.15 |
| 15 | chromatin silencing (GO:0006342) | 0.01105 | -2.61 |
| 16 | histone H3-K4 methylation (GO:0051568) | 0.01650 | -2.87 |
| 17 | embryo development (GO:0009790) | 0.007975 | -2.36 |
| 18 | histone H4 acetylation (GO:0043967) | 0.01105 | -2.29 |
| 19 | protein alkylation (GO:0008213) | 0.01105 | -2.27 |
| 20 | protein methylation (GO:0006479) | 0.01105 | -2.27 |
| 21 | macromolecule methylation (GO:0043414) | 0.01263 | -2.32 |
| 22 | regulation of type I interferon-mediated signaling pathway (GO:0060338) | 0.01604 | -2.45 |
| 23 | regulation of cell growth (GO:0001558) | 0.01286 | -2.30 |
| 24 | ATP catabolic process (GO:0006200) | 0.01503 | -2.29 |
| 25 | nucleoside monophosphate catabolic process (GO:0009125) | 0.01628 | -2.30 |
| 26 | purine nucleoside monophosphate catabolic process (GO:0009128) | 0.01604 | -2.29 |
| 27 | ribonucleoside monophosphate catabolic process (GO:0009158) | 0.01604 | -2.29 |
| 28 | regulation of sequence-specific DNA binding transcription factor activity (GO:0051090) | 0.01653 | -2.30 |
| 29 | purine ribonucleoside monophosphate catabolic process (GO:0009169) | 0.01604 | -2.28 |
| 30 | regulation of small GTPase mediated signal transduction (GO:0051056) | 0.01650 | -2.27 |
| 31 | regulation of cytoskeleton organization (GO:0051493) | 0.02105 | -2.41 |
| 32 | regulation of type I interferon production (GO:0032479) | 0.01538 | -2.22 |
| 33 | response to type I interferon (GO:0034340) | 0.01263 | -2.12 |
| 34 | negative regulation of gene expression, epigenetic (GO:0045814) | 0.02140 | -2.41 |
| 35 | cellular component morphogenesis (GO:0032989) | 0.02105 | -2.33 |
| 36 | Wnt signaling pathway (GO:0016055) | 0.01858 | -2.25 |
| 37 | regulation of actin cytoskeleton organization (GO:0032956) | 0.02105 | -2.32 |
| 38 | ATP metabolic process (GO:0046034) | 0.02105 | -2.30 |
| 39 | small GTPase mediated signal transduction (GO:0007264) | 0.02140 | -2.25 |
| 40 | positive regulation of type I interferon production (GO:0032481) | 0.02105 | -2.14 |
| 41 | in utero embryonic development (GO:0001701) | 0.02693 | -2.24 |
| 42 | regulation of mRNA metabolic process (GO:1903311) | 0.02088 | -2.09 |
| 43 | chordate embryonic development (GO:0043009) | 0.03076 | -2.25 |
| 44 | regulation of actin filament-based process (GO:0032970) | 0.03364 | -2.30 |
| 45 | purine ribonucleoside monophosphate metabolic process (GO:0009167) | 0.03432 | -2.28 |
| 46 | activation of MAPKK activity (GO:0000186) | 0.02160 | -2.00 |
| 47 | purine nucleoside monophosphate metabolic process (GO:0009126) | 0.03471 | -2.28 |
| 48 | embryo development ending in birth or egg hatching (GO:0009792) | 0.03364 | -2.25 |
| 49 | microtubule-based process (GO:0007017) | 0.03685 | -2.30 |
| 50 | regulation of Ras GTPase activity (GO:0032318) | 0.03685 | -2.26 |

**Supplementary Table 6B**: **GO biological processes affected by the genes with negative correlation to Spinophilin in Squamous cell lung tumors (TCGS database). Based on Gene list from Supplementary Table 3. Performed through Enrichr web page (http://amp.pharm.mssm.edu/Enrichr/enrich)**

| **Index** | **Name** | **Adjusted p-value** | **Z-score** |
| --- | --- | --- | --- |
| 1 | respiratory electron transport chain (GO:0022904) | 1,62E-09 | -2.17 |
| 2 | electron transport chain (GO:0022900) | 1,66E-09 | -2.15 |
| 3 | generation of precursor metabolites and energy (GO:0006091) | 1,68E-04 | -2.37 |
| 4 | gene expression (GO:0010467) | 1,70E-04 | -2.33 |
|   \| 5 \| \| --- \| | negative regulation of protein ubiquitination (GO:0031397) | 0.00001496 | -2.23 |
| 6 | cell cycle G1/S phase transition (GO:0044843) | 0.00001496 | -2.22 |
| 7 | G1/S transition of mitotic cell cycle (GO:0000082) | 0.00001496 | -2.22 |
| 8 | negative regulation of ubiquitin-protein ligase activity involved in mitotic cell cycle (GO:0051436) | 0.00001496 | -2.15 |
| 9 | regulation of ubiquitin-protein ligase activity involved in mitotic cell cycle (GO:0051439) | 0.00001496 | -2.15 |
| 10 | negative regulation of protein modification by small protein conjugation or removal (GO:1903321) | 0.00002800 | -2.20 |
| 11 | positive regulation of ubiquitin-protein ligase activity involved in mitotic cell cycle (GO:0051437) | 0.00002800 | -2.15 |
| 12 | negative regulation of ubiquitin-protein transferase activity (GO:0051444) | 0.00003394 | -2.17 |
| 13 | negative regulation of ligase activity (GO:0051352) | 0.00003394 | -2.17 |
| 14 | mitotic cell cycle (GO:0000278) | 0.00008375 | -2.27 |
| 15 | proteasome-mediated ubiquitin-dependent protein catabolic process (GO:0043161) | 0.00009362 | -2.25 |
| 16 | positive regulation of ligase activity (GO:0051351) | 0.00008375 | -2.21 |
| 17 | ATP synthesis coupled proton transport (GO:0015986) | 0.0004444 | -2.64 |
| 18 | energy coupled proton transport, down electrochemical gradient (GO:0015985) | 0.0004444 | -2.63 |
| 19 | regulation of ligase activity (GO:0051340) | 0.0001097 | -2.21 |
| 20 | regulation of protein ubiquitination (GO:0031396) | 0.0001289 | -2.24 |
| 21 | proteasomal protein catabolic process (GO:0010498) | 0.0001289 | -2.24 |
| 22 | anaphase-promoting complex-dependent proteasomal ubiquitin-dependent protein catabolic process (GO:0031145) | 0.00009722 | -2.16 |
| 23 | regulation of cellular amino acid metabolic process (GO:0006521) | 0.0001289 | -2.16 |
| 24 | modification-dependent protein catabolic process (GO:0019941) | 0.0003201 | -2.40 |
| 25 | positive regulation of ubiquitin-protein transferase activity (GO:0051443) | 0.0001579 | -2.19 |
| 26 | mitochondrial ATP synthesis coupled proton transport (GO:0042776) | 0.0006573 | -2.60 |
| 27 | modification-dependent macromolecule catabolic process (GO:0043632) | 0.0003711 | -2.40 |
| 28 | mitochondrial electron transport, NADH to ubiquinone (GO:0006120) | 0.0002270 | -2.21 |
| 29 | regulation of ubiquitin-protein transferase activity (GO:0051438) | 0.0002270 | -2.20 |
| 30 | ubiquitin-dependent protein catabolic process (GO:0006511) | 0.0004444 | -2.39 |
| 31 | ATP biosynthetic process (GO:0006754) | 0.0004444 | -2.38 |
| 32 | regulation of protein modification by small protein conjugation or removal (GO:1903320) | 0.0003637 | -2.24 |
| 33 | protein catabolic process (GO:0030163) | 0.0004781 | -2.29 |
| 34 | proteolysis involved in cellular protein catabolic process (GO:0051603) | 0.0009149 | -2.38 |
| 35 | mitochondrial transport (GO:0006839) | 0.0004444 | -2.12 |
| 36 | purine ribonucleoside triphosphate biosynthetic process (GO:0009206) | 0.0006402 | -2.13 |
| 37 | purine nucleoside triphosphate biosynthetic process (GO:0009145) | 0.0007226 | -2.10 |
| 38 | DNA damage response, signal transduction by p53 class mediator resulting in cell cycle arrest (GO:0006977) | 0.001005 | -2.14 |
| 39 | signal transduction involved in mitotic G1 DNA damage checkpoint (GO:0072431) | 0.001105 | -2.14 |
| 40 | intracellular signal transduction involved in G1 DNA damage checkpoint (GO:1902400) | 0.001105 | -2.14 |
| 41 | signal transduction involved in mitotic cell cycle checkpoint (GO:0072413) | 0.001158 | -2.14 |
| 42 | signal transduction involved in mitotic DNA damage checkpoint (GO:1902402) | 0.001158 | -2.13 |
| 43 | signal transduction involved in mitotic DNA integrity checkpoint (GO:1902403) | 0.001158 | -2.13 |
| 44 | mitotic cell cycle phase transition (GO:0044772) | 0.001539 | -2.21 |
| 45 | signal transduction involved in DNA integrity checkpoint (GO:0072401) | 0.001401 | -2.12 |
| 46 | mRNA processing (GO:0006397) | 0.002075 | -2.25 |
| 47 | cell cycle phase transition (GO:0044770) | 0.001789 | -2.20 |
| 48 | nucleoside triphosphate biosynthetic process (GO:0009142) | 0.001158 | -2.06 |
| 49 | signal transduction involved in DNA damage checkpoint (GO:0072422) | 0.001401 | -2.12 |
| 50 | regulation of cellular amine metabolic process (GO:0033238) | 0.001177 | -2.05 |
